# Reinforcement of Colonic Anastomosis with Improved Ultrafine Nanofibrous Patch: Experiment on Pig

**DOI:** 10.3390/biomedicines9020102

**Published:** 2021-01-21

**Authors:** Jachym Rosendorf, Marketa Klicova, Lenka Cervenkova, Jana Horakova, Andrea Klapstova, Petr Hosek, Richard Palek, Jan Sevcik, Robert Polak, Vladislav Treska, Jiri Chvojka, Vaclav Liska

**Affiliations:** 1 Biomedical Center, Faculty of Medicine in Pilsen, Charles University, 301 00 Pilsen, Czech Republic; lenka.cervenkova@lfp.cuni.cz (L.C.); petr.hosek@lfp.cuni.cz (P.H.); palekr@fnplzen.cz (R.P.); sevcik.jan97@seznam.cz (J.S.); polakr@fnplzen.cz (R.P.); 2 Department of Surgery, Faculty of Medicine in Pilsen, Charles University, 301 00 Pilsen, Czech Republic; treska@fnplzen.cz; 3 Department of Nonwovens and Nanofibrous Materials, Faculty of Textile Engineering, Technical University of Liberec, 460 01 Liberec, Czech Republic; marketa.klicova@seznam.cz (M.K.); horakova2222@gmail.com (J.H.); a.klapstova@centrum.cz (A.K.); jiri.chvojka@tul.cz (J.C.)

**Keywords:** colorectal surgery, nanofibrous materials, anastomotic leakage, intestinal anastomosis, anastomotic patch, polycaprolactone, electrospinning, experiment, peritoneal adhesions

## Abstract

Anastomotic leakage is a dreadful complication in colorectal surgery. It has a negative impact on postoperative mortality, long term life quality and oncological results. Nanofibrous polycaprolactone materials have shown pro-healing properties in various applications before. Our team developed several versions of these for healing support of colorectal anastomoses with promising results in previous years. In this study, we developed highly porous biocompatible polycaprolactone nanofibrous patches. We constructed a defective anastomosis on the large intestine of 16 pigs, covered the anastomoses with the patch in 8 animals (Experimental group) and left the rest uncovered (Control group). After 21 days of observation we evaluated postoperative changes, signs of leakage and other complications. The samples were assessed histologically according to standardized protocols. The material was easy to work with. All animals survived with no major complication. There were no differences in intestinal wall integrity between the groups and there were no signs of anastomotic leakage in any animal. The levels of collagen were significantly higher in the Experimental group, which we consider to be an indirect sign of higher mechanical strength. The material shall be further perfected in the future and possibly combined with active molecules to specifically influence the healing process.

## 1. Introduction

Anastomotic leakage (AL) is a severe and feared complication in colorectal surgery. There used to be a lack of consensus over the classification of such conditions in the past, making it difficult to compare complication rates after specific types of procedures. Rahbari et al. [1] created a clear classification of the leaks depending on the type of approach to the complication, which is generally accepted by the wider medical community. However, different hospitals have different approaches and what could be treated conservatively in one department (classified as grade A or B [1]), could also end up with an anastomosis resection and a Hartmann procedure in another (classified as grade C [1]). It is therefore very difficult to assess the real incidence of AL, however it is usually reported to be as high as 5 to 19% [2,3,4]. The majority of colorectal procedures are performed for colorectal cancer and the number of performed procedures is enormous. Therefore, these complications form a great medical problem [5,6].

Many risk factors have been identified and one of the strongest is the position of anastomosis. Especially low anastomoses (within 5 or 6 cm from the anal verge [7,8]) show high risk of AL [9,10]. Other known factors are age, gender, smoking, steroid therapy and more [9,10,11]. All of the risk conditions are assumed to decrease the patient’s healing abilities generally or locally. However, the specific pathophysiological mechanisms are not well described. As postoperative life quality is often terribly compromised after such complications and the complication itself is in many cases (especially grade C) fatal, AL is considered a large socioeconomic burden [12,13].

Peritoneal adhesions (PAs) are a common problem in abdominal surgery. They are formed in various extents after all surgical procedures and also other damage to the peritoneal cavity. Their purpose is protective, however they are in many cases a source of long term postoperative complications such as gastrointestinal obstruction, infertility or abdominal discomfort [14].

Some kind of patch seems to be a promising solution for local prevention of AL (and possibly PAs). Many materials have been tested for these purposes yet none of them are currently accepted in routine clinical practice [15,16,17]. There also has not been any material developed and tested for prevention of both PAs and AL according to our knowledge and a literature search.

Nanofibrous materials are nonwoven fabrics created by different techniques, usually from polymeric biomaterials. The variety of source materials and range of fabrication protocols offer an enormous spectrum of such fabrics, naturally resulting in novel applications in medical use. Some versions of nanofibrous planar biodegradable materials have been described to have a positive effect on wound healing by several authors [18,19,20]. It is assumed to be caused, among other factors, by its structural similarities to collagenous extracellular matrix [19]. There are a variety of synthetic biodegradable materials suitable for fabrication of nanofibrous scaffolds such as polycaprolactone (PCL), polylactide, polyglycolide, polydioxanone, polyhydroxybutyrate and others [21]. PCL is among the most used for implantable devices because of its good mechanical and biological properties and for the fact that it is a substance already in use in clinical medicine [22,23,24].

Electrospinning is one of the most commonly used approaches for scaffold production. The versatility of the process together with easily controlled parameters has led to wide use of electrospun scaffolds in the field of regenerative medicine and tissue engineering [25]. In our study, the planar nanofibrous PCL layers were fabricated via a needleless electrospinning technique called Nanospider^TM^. The chosen method contrasts with commonly used needle electrospinning by allowing large-scale industrial production, thus supporting further introduction of the material to the market.

Our team developed and tested several versions of these materials [26,27]. A complex histological, clinical and macroscopic evaluation system has been perfected in recent works [26].

The healing process of both a skin wound or an anastomosis on the small or the large intestine is a complicated process that is yet to be fully explored and understood [28]. However, some parts of the process are known and it is certain that this process must remain well balanced for a successful outcome. A healthy peritoneum is a well-perfused metabolically active structure capable of relatively high metabolic exchange with its surroundings including both peritoneal fluid and other viscera and neighboring peritoneal surfaces [29,30]. Based on the results of our previous experiments and on the presumption that a certain level of metabolic exchange between the sutured intestine and the surrounding peritoneal surfaces is needed to maintain the healing process rather than creating a sealed barrier, we decided to create a very fine porous nanofibrous patch. Such a patch should allow this metabolic exchange while maintaining the pro-healing properties of a nanofibrous mesh we proposed in the previous studies [26,27]. The process conditions for fabricating a material with a low surface density were optimized via needleless electrospinning.

According to our knowledge, our study is the first to propose the idea of a porous anastomotic patch for healing support that should not act only as a mechanical barrier, but support the healing process of the intestinal anastomosis. We intend to develop such a patch into a product that could be routinely used in colorectal surgery for healing support in either all or high risk anastomoses.

In this study we aimed to develop an ultrafine porous polycaprolactone nanofibrous patch, use it in a perfected model of complicated anastomotic healing on the large intestine, and further develop current assessment methods for evaluation of anastomotic healing in experimental settings.

## 2. Materials and Methods

### 2.1. Material Preparation (Electrospinning Method) 

A mixture of 16% w/w PCL (Mw 45,000 g/mol, Sigma Aldrich, St. Louis, MI, USA) in chloroform/ethanol/acetic acid in ratio 8/1/1 (Penta Chemicals, Prague, Czech Republic) was stirred 24 h until complete dissolution of the PCL granulate. Subsequently, the solution was electrospun using the needleless NanospiderTM 1WS500U electrospinning device (Elmarco, Liberec, Czech Republic) (scheme in Supplementary Figure S1). The environmental parameters such as the relative humidity and temperature were controlled via the climatic system NS AC150 (Elmarco). The nanofibers were collected on a polypropylene spunbond substrate. The process parameters were optimized to produce a nanofibrous layer with low surface density, namely 10 g/m^2^ (listed in Appendix A, Table A1).

### 2.2. Material Characterization 

A scanning electron microscope (SEM) VEGA 3 TESCAN (SB Easy Probe, Brno, Czech Republic) was used to obtain the surface morphology of the fabricated nanofibers. Prior to scanning, the samples were sputter coated with 10 nm of gold using QUORUM Q50ES (Quorum technologies, Lewes, UK). The fiber diameters were assessed by the software IMAGE J (NIH Image, Bethesda, MD, USA) by randomly measuring 500 fibers in the scans. The specific weight was calculated by weighing of samples in the dimension 10 × 10 cm (*n* = 10).

Sterilization and in vitro biocompatibility tests: Before in vitro testing, the materials were sterilized via low temperature ethylene oxide (Anprolene, Andersen Sterilizers, Haw River, NC, USA) according to the Czech norm CSN EN ISO 11135-1. The materials were tested one week after sterilization to eliminate the effect of ethylene oxide residues in the layers. The PCL scaffolds were seeded with 3T3 mouse fibroblasts (ATCC, Manassas, VA, USA) in a concentration 7 × 10^3^ cells per well. Metabolic activity was evaluated after 3, 7, 14 and 21 days via colorimetric Cell Counting Kit-8 (CCK-8) (Dojindo Laboratories, Rockville, MD, USA). During the CCK-8 assay, the scaffolds were incubated with 10% (*v/v*) of CCK-8 solution in full DMEM media for 3 h at 37 °C, 5% CO_2_. Absorbance was measured at 450 nm (*n* = 5). The morphology of the cells on the PCL materials was also monitored. Fluorescence imaging was performed with Nikon Eclipse-Ti-E (Nikon Imaging, Prague, Czech Republic) on fixed cells with 2.5% *v/v* glutaraldehyde (Sigma Aldrich, St. Louis, MI, USA) in PBS by adding DAPI (for cell nuclei visualization) and phalloidin-FITC (for staining actin cytoskeleton) after 3, 7, 14 and 21 days. The MATLAB software (MATLAB Student R2020b, Mathworks, Natick, MA, USA) was used to calculate the number of cells per 1 mm^2^ of the scaffold from 10 random fields of view. Dehydrated samples with fixed cells were also scanned via SEM during the same time period to obtain the morphology of the cells.

### 2.3. Experimental Design 

**Figure 1:**
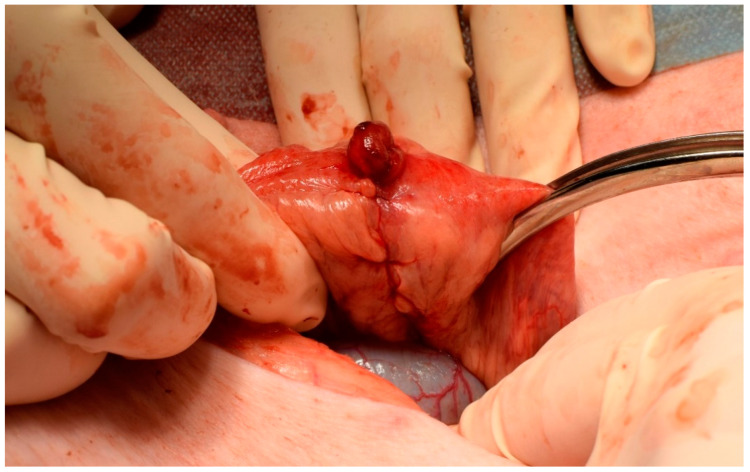
Construction of a defective anastomosis. Intestinal anastomosis with a defect on antimesenteric side pulled through a small incision.

We used 16 Prestice black-pied pigs in two groups; this number was chosen after consultation with a statistician (Supplementary Document S1). The animals were subjected to transection of the descending colon and anastomosis with a standardized defect under general anesthesia (Figure 1).

The defect was covered with the nanomaterial in the Experimental group while it was left uncovered in the Control group. The animals were observed for 3 weeks. Sample collection and macroscopic evaluation were performed on the 21st postoperative day (POD). Histological evaluation followed.

### 2.4. Surgical Procedure 

The animals were not fed on the day of the surgery, but no further intestinal preparation was applied. They were premedicated with ketamine (Narkamon 100 mg/mL, BioVeta a.s., Ivanovice na Hané, Czech Republic) and azaperone (Stresnil 40 mg/mL, Elanco AH, Prague, Czech Republic) administered intramuscularly. The animals were weighed prior to the surgical procedure. General anesthesia was maintained by continual application of propofolum MCT/LCT (Propofol 2% MCT/LCT Fresenius Medical Care a.s.). Nalbuphin (Nalbuphin, Torrex Chiesi CZ s.r.o., Prague, Czech Republic) was used for analgesia. A single dose of 0.6 g Amoksiklav (Amoksiklav 1.2 g, Sandoz s.r.o., Prague, Czech Republic) was administered intravenously 30 min before the skin incision, a second 0.6 g dose was administered 2 h later.

A Pro-Port implantable central venous catheter (Deltec, Smiths medical, Minneapolis, MN, USA) was introduced in general anesthesia through the right jugular vein and attached to the subcutaneous tissue on the right lateral side of the neck in each animal for easy and stress-less manipulation with the animal during the follow-up. After the implantation, we entered the abdominal cavity via a 10-cm-long transrectal incision performed in the left caudal abdominal quadrant. We pulled the descending colon up through the incision. We then transected the colon approximately 20 cm from the anus. We used soft intestinal clamps to prevent solid intestinal contents from contaminating the abdominal cavity. We cleaned the two ends of the transected colon using wet cotton balls. We constructed a hand-sewn end-to-end anastomosis using the standard seromuscular running suture using glyconate monofilament 4/0 suture line (Monocryl 4/0, B. Braun Medical s.r.o., Prague, Czech Republic). We intentionally left a 1-cm-large defect on the ventral side of the anastomosis, simulating a technical fault. We placed a standard 2.5-cm-wide sheet of the nanomaterial onto the sutured intestine, covering the intestinal circumference with the defect and the neighboring parts of the mesocolon in the Experimental group. We left the defect uncovered in the Control group. We placed the colon back to the abdominal cavity and sutured the peritoneum with an absorbable material (Vicryl 3/0, Ethicon Inc., Johnson & Johnson, s.r.o., Prague, Czech Republic) to prevent adhesions to the abdominal wall. Then we closed the muscle layer using single non-absorbable sutures (Mersilene 1, Ethicon Inc., Johnson & Johnson, s.r.o., Prague, Czech Republic). We rinsed the subcutaneous tissue with saline solution before finally suturing the skin.

### 2.5. Postoperative Observation 

The animals were observed for 3 weeks and they were checked daily for stool passage, body temperature and clinical signs of complications by both a surgeon and a veterinarian. Activity of the animals was scored using a 4-point scale (normal activity, decreased activity, little to no activity, irritated animal). Intravenous infusions of 250 mL 10% glucose and 250 mL Hartmann solution were applied daily in the first 3 Postoperative days (PODs). The animals were fed according to a re-alimentation schedule created for previous experiments. When feeding intolerance occurred, intravenous infusions were administered in the same way as in the first three PODs. Blood samples were obtained in defined time points (before the surgical procedure, 2 h after construction of colonic anastomosis, on the 1st POD, 3rd POD, 7th POD, 14th POD, 21st POD) and tested for blood count, level of bilirubin, liver enzymes, hemoglobin, urea and creatinine to distinguish metabolic disorders. Animals were weighed each time the blood sample was taken. A 5% weight difference from the initial weight was considered a significant weight change.

### 2.6. Macroscopic Evaluation 

The animals were subjected to laparotomy again on the 21st POD under general anesthesia. The abdominal cavity was inspected and checked for signs of AL (visible free intestinal contents or purulent secretion, macroscopic changes of peritoneal surfaces), visible defects in the site of anastomosis, changes in the intestinal diameter (stenosis of the anastomosis, dilation of oral segments of the intestine) or any other visible postoperative changes. At same time, the extent and location of PAs (according to qualitative Zühlke’s grading and quantitative Peritoneal Adhesions Amount Score (PAAS) (Supplementary Figure S2) [26]), amount and macroscopic quality of peritoneal fluid and the position and appearance of the nanofibrous material (if present) were recorded.

The intestinal specimens including the anastomoses were collected together with surrounding adhering tissues, cut on the mesenteric side longitudinally, pinned onto a cork underlay and stored in 10% buffered formalin.

### 2.7. Histological Evaluation 

The intestinal samples were cut into 5 pieces, 5 mm thick, crosswise to the line of the anastomosis in the area of the anastomotic defect. The tissues were processed by common paraffin technique. Each sample was cut to 5 µm slides and stained with hematoxylin and eosin for comprehensive overview; a Gomori trichrom kit was used to stain connective tissues.

The samples were investigated semi-quantitatively and quantitatively. Epithelization, inflammatory infiltration and necrosis were assessed in a single overall semi-quantitative investigation (Intestinal Wall Integrity Score (Appendix B, Table A2)). The inflammatory reaction to stitches and microabscesses were not included in the score. The score was determined for all five blocks, and the three blocks with the highest score (corresponding to the area of the anastomotic defect) were used for statistical evaluation.

The blocks with the highest total score for each pig were subsequently analyzed quantitatively; 5 µm sections were stained with picrosirius red (Direct red 80) for visualization of collagen in polarized light. Immunohistochemical methods were used for detection of the vascular endothelium using Anti-Von Willebrand Factor antibody (Abcam ab6994, dilution 1:400); Calprotectin Monoclonal Antibody MAC387 (Invitrogen MA1-81381, dilution 1:200) was used for detection of granulocytes and tissue macrophages.

The area for quantitative evaluation for samples without visible defect of the muscular layer was defined as the intestinal wall excluding mucosa located 3 mm orally and aborally from the center of the anastomosis. The evaluation area for samples with a defect of the muscular layer or pseudodiverticulum was defined as 2 mm orally and aborally from the defect margins. The volume of endothelial cells, volume of MAC387 positive cells and volume of collagen was assessed using stereological methods in a similar way as in a previous study [26].

### 2.8. Statistics 

Common descriptive statistics and frequencies were used to characterize the sample data set. Due to their non-normal distribution, the intestinal wall integrity scores and histologically determined volume fractions were compared between the Experimental and Control group using Mann–Whitney U test in STATISTICA data analysis software (Version 12, StatSoft, Inc., Tulsa, OK, USA). The material properties, presented as mean ± standard deviation (SD), were analyzed using GraphPad Prism (Version 7, GraphPad Software, San Diego, CA, USA). Firstly, the Shapiro–Wilk test was used to prove or reject the normal distribution of the data. For the normally distributed data, a parametric ANOVA test with Tukey’s multiple comparison was performed. The nonparametric Kruskal–Wallis with Dunn’s multiple comparison was chosen for the data following non-normal distribution. All reported *p* values are two-tailed and the level of statistical significance was set at α = 0.05.

## 3. Results

### 3.1. Material Properties

**Figure 2:**
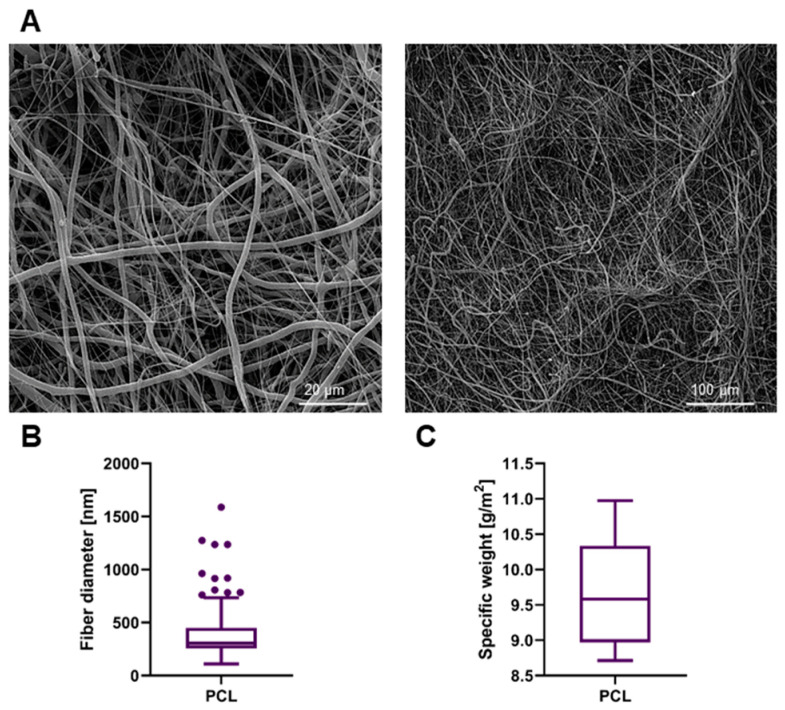
The SEM (scanning electron microscopy) images of the electrospun PCL (polycaprolactone) planar layer, scale bars 20 µm and 50 µm (**A**). The boxplot of fiber diameters (*n* = 500) (**B**). The calculated value of specific weight of the nanofibrous layer (*n* = 10) (**C**).

Sheets of PCL nanofibrous material were successfully prepared and sterilized. The material appeared very subtle yet the manipulation with it was still comfortable. The material was easy to apply onto the intestinal surface and it remained adhered to the spot of application without any need of further fixation. The morphology of the fibrous material was assessed by SEM (Figure 2A). The fibers had no defects and were without any dominant orientation. The fiber diameter was (385 ± 239) nm (Figure 2B). The high SD is a consequence of ultrafine fibers being present together with larger ones. The specific weight of the material was calculated as (9.67 ± 0.77) g/m^2^; the data are symmetrical around the mean value (Figure 2C).

### 3.2. Cytocompatibility

**Figure 3:**
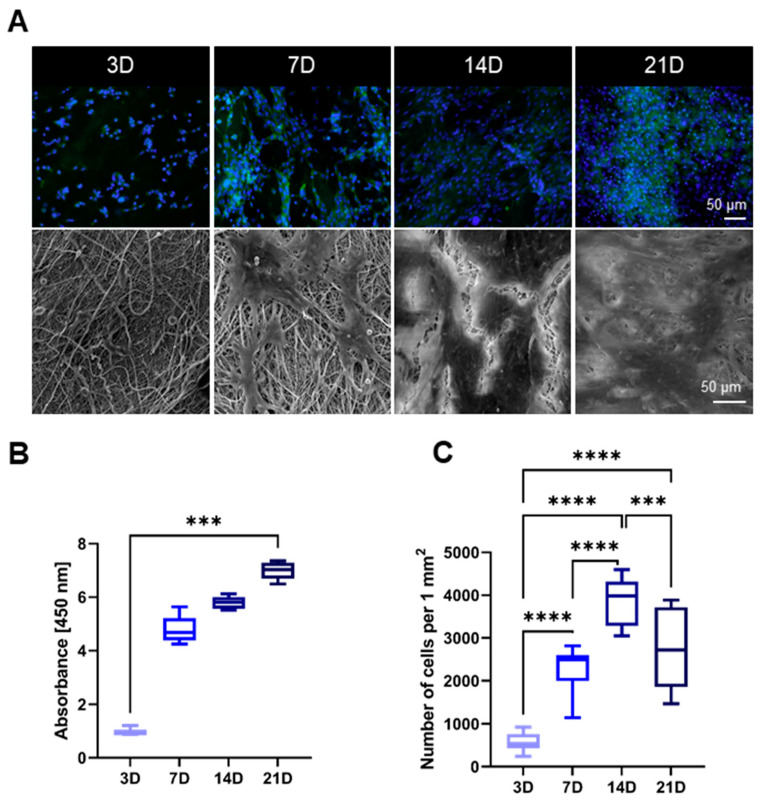
Fluorescence microscopy images (blue cell nuclei and green actin cytoskeleton) and SEM images of the cells on the PCL scaffold after 3, 7, 14 and 21 days of the in vitro testing, scale bars 50 µm (**A**). The result of the colorimetric CCK-8 assay after the same time period, Kruskal–Wallis *** *p* = 0.0004. (**B**). Counted number of the cells on the surface of PCL materials per 1 mm^2^, ordinary one-way ANOVA, *** *p* < 0.0006, **** *p* = 0.0001 (**C**).

Adhesion, proliferation and morphology of the 3T3 mouse fibroblasts on the PCL scaffolds were monitored with fluorescence microscope and the scanning electron microscope after 3, 7, 14 and 21 days (Figure 3A). The length of the experiment corresponds with the duration of the in vivo study. Cell viability was determined using a colorimetric assay CCK-8 after 3, 7, 14 and 21 days of incubation of 3T3 mouse fibroblasts with the tested fiber layers. The obtained mean absorbance values express the cell viability of the cultured cells (Figure 3B). According to the CCK-8 assay, the absorbance was low during the first testing day, which is in positive correlation with the microscopy observation. On the seventh day of cultivation, an increase in viability was measured. At the same time, spreading of the cells was observable on the microscopy images, as the cells expanded across the material and began to form isolated cell islands. After 14 days of cultivation, there was a further increase in viability and the cells formed a sub-confluent layer. On the last testing day, the SEM image revealed 100% confluence of the cells. The number of the cells (Figure 3C) correlates with the remaining results. The highest cell density was observed during the 14th day (3887 ± 539) cells/mm^2^, while on the last testing day it dropped to (2735 ± 880) cells/mm^2^.

### 3.3. Manipulation

The material was easy to apply and no further fixation was needed. Procedure times were not prolonged by the usage of the material.

### 3.4. Clinical Results

All animals survived the observation period in good clinical condition. A temporary activity decrease was observed in one animal from the Control group (12.5%) and in three animals from the Experimental group (37.5%).

There were no major complications during the observation period. Laparotomy wound infection occurred in one animal from the Experimental group (12.5%) and one animal from the Control group (12.5%). Infection of the skin wound of the pro-port system occurred in the same animal from the Control group (12.5%).

No animal developed signs of gastrointestinal obstruction (vomiting, feeding intolerance). No animal developed signs of peritonitis and sepsis (abdominal wall tenderness, significant activity decrease, significant laboratory changes). Peroral intake was tolerated by all animals, all animals were fed according to the schedule with no exceptions. Only three animals from the Control group (37.5%) gained more than 5% of weight during the experiment, while six animals from the Experimental group (75%) showed such weight gain (Appendix C, Table A3).

### 3.5. Macroscopic Results

**Figure 4:**
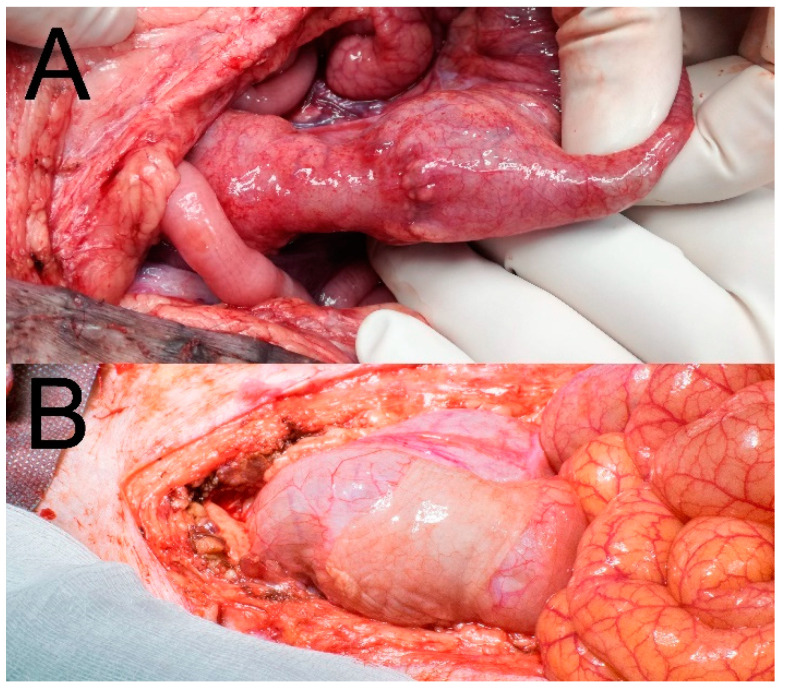
Macroscopic findings in situ at the end of the observation period; (**A**) stenotic anastomosis from the Control group; (**B**) anastomosis with attached material (Experimental group).

There was no macroscopically visible pathological reactions to the material in theabdominal cavities of the animals after 3 weeks of observation. Four animals (50%) had no PAs at the site of the anastomosis in the Control group, while three animals (37.5%) from the Experimental group had no PAs there. A mean PAAS value of 1 was recorded in both the Control and the Experimental group (Tab). All PAs were scored 2 points according to the Zühlke’s grading system in both groups (partially vascularized adhesions, possible to separate by combination of blunt and sharp dissection). Stenosis of the anastomosis was observed in one animal from the Control group (12.5%) with low shrinkage of the intestinal diameter (less than 1/3) (Figure 4A). No stenoses were observed in the Experimental group (Figure 4B). No signs of gastrointestinal obstruction (dilatation of oral segments) were observed in any of the animals. No macroscopic signs of AL were observed (no visible defect in the site of the colonic anastomosis, no free intestinal content in the abdominal cavity).

**Figure 5:**
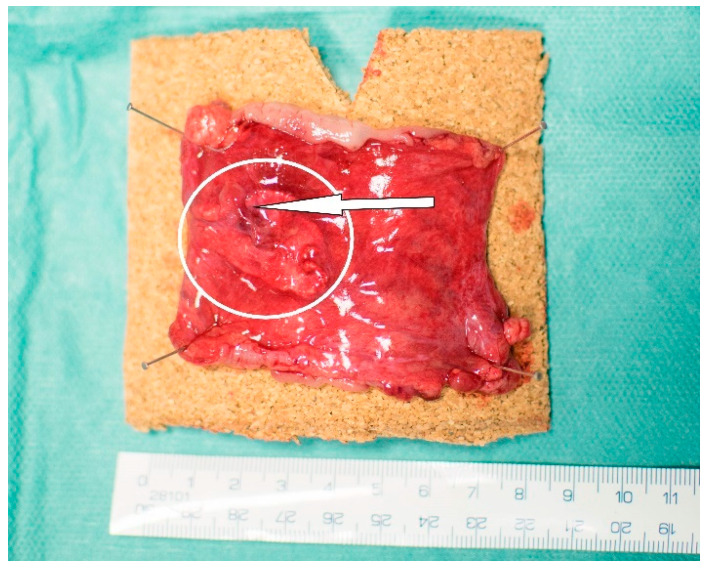
A specimen from the Experimental group prepared for fixation. Partial dislocation of the material (circled), residue of a PA (arrow).

**Figure 6:**
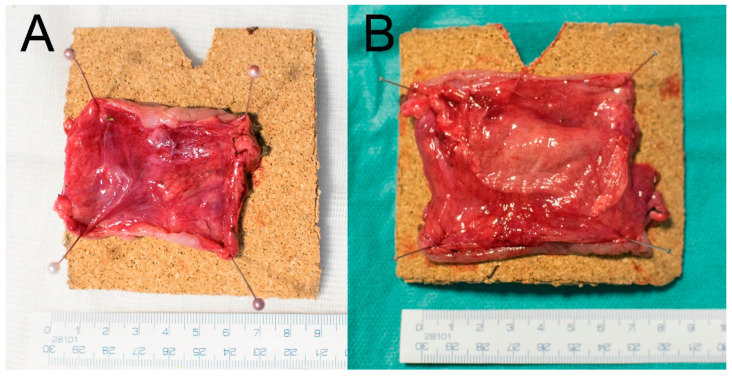
Specimens prepared for fixation. (**A**) A specimen from the Control group; (**B**) a specimen from the Experimental group. The material covers the line of anastomosis well.

Complete dislocation of the material was not observed in any of the animals of the Experimental group. Partial dislocation was observed in three animals (37.5%), however the material always kept covering the location of the anastomotic defect (Figure 5). The defect was not visible in the Control specimens without a patch (Figure 6A). The material was well attached in the most of the specimens (Figure 6B).

Most of the adhesions in the site of the anastomosis were between the large intestine and the urinary bladder. There were no PAs observed in the rest of the abdominal cavity in any animal.

### 3.6. Blood Sample Results

There were no statistically significant differences in the measured parameters between the two groups and no significant deviations from normal levels of the parameters (see Supplementary Table S1).

### 3.7. Histological Results

**Figure 7:**
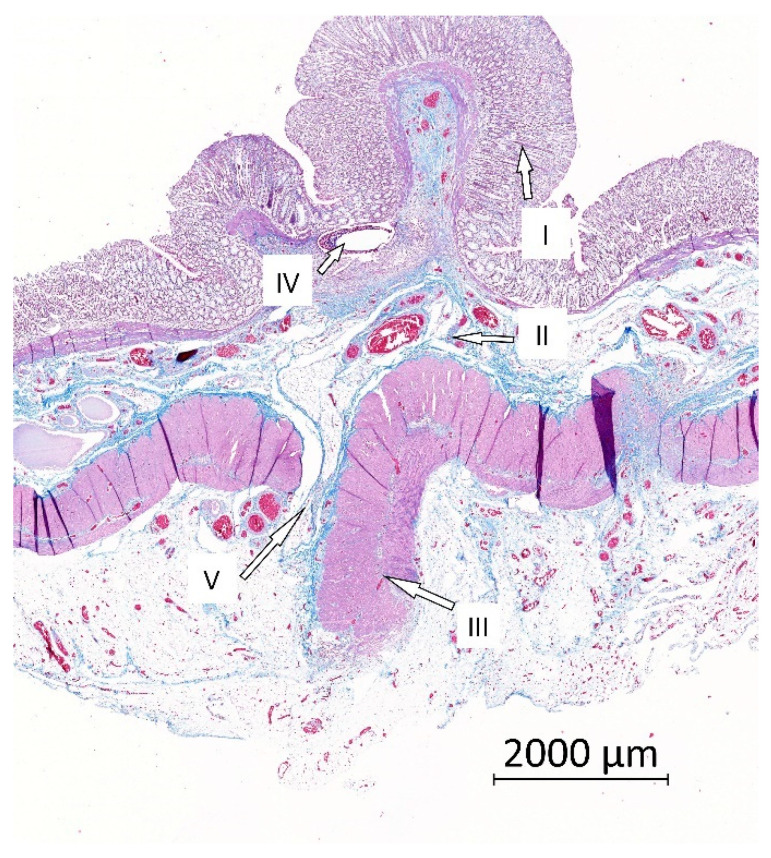
Example histological specimen from the Control group, Gomori trichrome staining. (I) The mucosa; (II) the submucosa; (III) the muscular layer; (IV) a defect after suture material that was washed out during histological processing; (V) location of the anastomosis with normal scar tissue.

**Figure 8:**
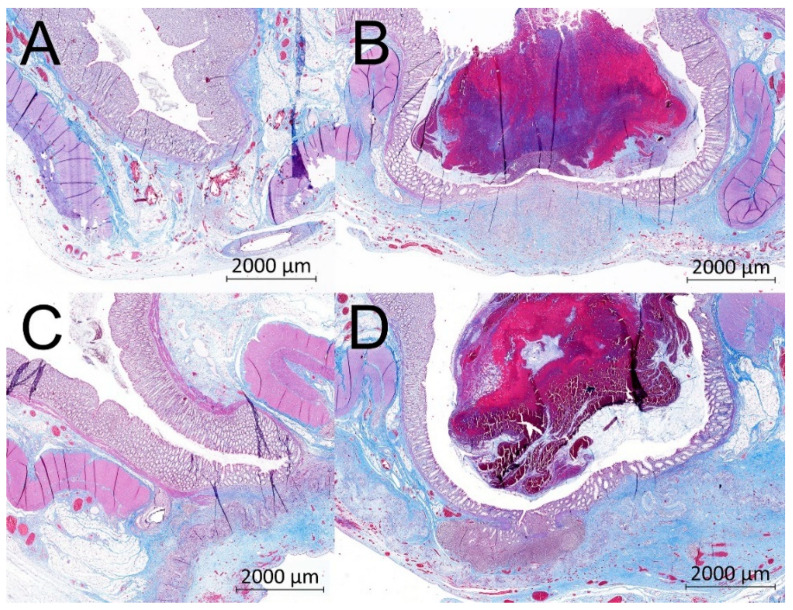
Example histological specimens from both groups. Gomori trichrome (**A**) Control group, optimal healing, normal morphology of the intestinal wall, muscular layer with normal scar tissue; (**B**) Control group, larger defect of the muscular layer, a pseudodiverticulus; (**C**) Experimental group, optimal healing, normal morphology of the intestinal wall, visible residues of the nanofibrous material in the bottom of the image; (**D**) Experimental group, large defect of the muscular layer, a pseudodiverticulus, visible residues of the nanofibrous material in the bottom of the image covering the incomplete defect of the intestinal wall.

**Figure 9:**
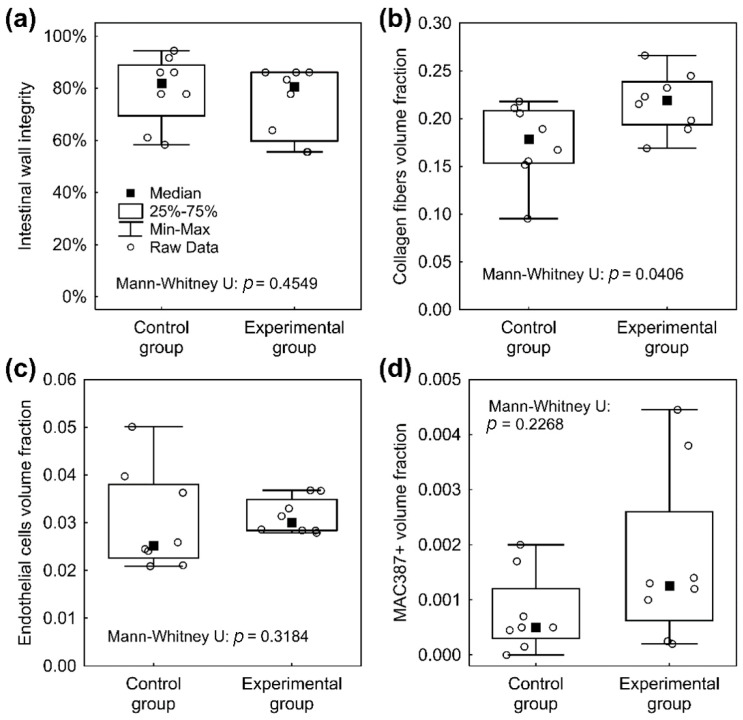
Graphical depiction of main histological results. (**a**) The intestinal wall integrity score in the Control group and the Experimental group with no significant differences with median value above 80%. (**b**) Significantly higher volume fractions of collagen at the site of anastomosis in the Experimental group. (**c**) No significant differences between the two groups in volume fractions of endothelial cells, lower dispersion range of values in the Experimental group. (**d**) The difference in volume fractions of inflammatory cells at the site of anastomosis between the groups is not statistically significant.

The material was washed out during the histological fixation and staining. There were no microscopic signs of AL (no full-thickness defect was found in any specimen in either the Control or the Experimental group). We found normal morphology of the intestinal wall in all specimens using a comprehensive overview (Figure 7). In some cases, the muscular layer did not heal completely and pseudodiverticula were formed (three cases in the Control group (37.5%) and seven cases (87.5%) in the Experimental group; Figure 8)). There was no statistically significant difference between the groups according to our Intestinal Wall Integrity Score (Figure 9A). There were significantly higher volume fractions of collagen in the Experimental group (Figure 9B). There was no statistically significant difference between the two groups in volume fractions of MAC 387 positive cells (Figure 9D) and endothelial cells (Figure 9C).

## 4. Discussion

We developed a nanofibrous material based on biodegradable polycaprolactone with very low specific weight. The material was uniquely designed for the reinforcement of GI anastomoses and its design was based on our previous in vitro and in vivo experiments. Polycaprolactone is often used for its biocompatibility and biodegradability [31,32,33]. The in vitro testing with 3T3 mouse fibroblasts proved the cytocompatibility of the material; the cells formed a fully confluent layer on the surface of the scaffold after 21 days. This observation is consistent with other literature resources, where the combination of micro- and nanofibers in PCL scaffolds supported cell growth [21,22]. Prior to the in vitro testing, the scaffolds were sterilized with low temperature ethylene oxide with respect to the low melting point of PCL. The possible effect of the ethylene oxide sterilization on PCL was already examined in our previous study by Horakova et al. [27]. The PCL patches are easy to apply and we value this as an important property. While the material is very subtle as its specific weight is only 10 g/m^2^, it was still mechanically strong enough to be handled easily. The material always remained in the site of application during the surgical procedure and during reposition of the viscera without further fixation. The convenient application together with natural fixation are key properties should this approach be used in routine clinical practice.

We successfully created a model of anastomosis with a defect on the large intestine of a pig. We used the Testini’s [16] modified model from previous experiments [26] in order to move the anastomosis to a location with bacterial contamination and higher risk of healing complications. The defect was chosen to be small enough to simulate a technical fault (which is also one of the contributing factors of AL [34]) and large enough to induce imperfect healing. The position of the anastomosis 20 cm from the anus was chosen for its good accessibility, no need for further preparation, possibility of small abdominal wound and therefore low non-anastomosis related complication risk. The model allowed us to focus only on imperfect anastomotic healing with no other disturbing factors. Together with the assessment methodology, the model allowed a reduction in the number of experimental animals and gave what we consider statistically reliable results. A three-week observation period was chosen based on our previous experience and the possibility of using evaluation histologic systems from previous publications. AL is typically an early complication, usually appearing within the first 10 PODs [2,35]. To verify the behavior of the material in a long term period regarding its complete absorption and impact on the risk of late complications, longer observation times would be necessary.

All of the animals in both groups survived the observation period in good clinical shape with a low complication rate. An activity decrease was observed only in the early postoperative period in both groups, which we considered as normal postoperative state. The feeding tolerance was equally good in both groups. The animals from the Experimental group gained weight in more cases than in the Control group. Weight gain is a sign of good postoperative adaption [36]. No animal developed ileus or sepsis or other serious pathological reaction to the material. This contributes to our assumption that it is safe to use in this application.

We observed slight shifting of the material in a few cases, however the material always remained covering the spot of anastomotic defect. We observed this also in the last study on the small intestine with an earlier version of the material, and therefore we assume it is not a coincidence [26]. This barrier was always present even in specimens with larger defect of muscular layer, and no macroscopic or microscopic AL was observed. It remains a question whether the material is able to prevent manifestation of AL. An anastomotic leakage is in experiments usually obtained by either large anastomotic defects or other negative influences (infection, radiation, devascularization). The model of a small defect was chosen to study the impact of the material on imperfect anastomotic healing in highly standardized conditions.

There was one partial shrinkage of the intestinal diameter at the site of the anastomosis in one animal from the Control group (12.5%), therefore we assume the material does not cause formation of anastomotic strictures. Those can however develop in longer time periods and thus a longer observation time would be needed to verify this information [37].

The level of adhesions was similarly low in both groups, suggesting the current material version to be the first in our series of polycaprolactone electrospun materials without pro-adhesive properties [26]. We consider the generally low amount of adhesions to be also a result of short procedure times with low manipulation with tissues [38]. Excessive formation of PAs is considered to be a result of a healing problem [39]. The visceral peritoneum is the superficial layer of the intestine, so wound healing of the peritoneum is a part of anastomotic wound healing. Therefore, we think, qualitative and quantitative assessment of PAs should be involved in the evaluation of anastomotic healing [26].

There were no statistically significant differences in vascularization and inflammatory cells infiltration according to the stereological measurements. This suggests a normal healing process [40]. However, the levels of collagen were found higher in the Experimental group. It was previously observed in mechanical tests of intestinal anastomoses that higher levels of collagen are associated with higher mechanical strength and higher anastomotic bursting pressure [41]. Bacterial collagenases were identified as a possible contributor to development of AL. Their activity causes collagen degradation in the site of intestinal anastomosis. Intestinal colonization with several bacterial species was identified as a strong risk factor of AL due to their production of collagenases [41,42,43].

We used both traditional evaluation methods [40] with those that were developed for our purposes in previous papers [26]. The intestinal wall integrity score from the previous study was adjusted for a defective model on the large intestine. Together with the rest of the involved assessment methods, it forms the most robust and complex evaluation system of anastomotic healing in similar experiments according to our knowledge and literature search.

The above-mentioned results all suggest possible contribution to AL prevention by our material only indirectly. To obtain more distinguishable results, a model with more compromised anastomotic healing with high risk of AL manifestation would be necessary. This is certainly a limitation of this study.

Because the material was washed out during the histological processing, we cannot evaluate the level of biodegradation. However, this was studied earlier for PCL in other forms [44].

The material seems to be an ideal version for use in combination with active substances like anti-inflammatory drugs, antibacterial agents or antibiotics as an anastomotic patch. Polycaprolactone was identified as a good medium for regulated drug release [33,45]; there is a broad spectrum of active molecules that could be beneficial for either AL prevention or prevention of excessive PA formation [39,46,47,48,49]. Therefore, we intend to perfect the material using these substances and to study their impact on anastomotic healing and complications further to finally offer a perfect anastomotic patch for patients with high risk of AL. Possible clinical studies will be planned afterwards.

## 5. Conclusions

We succeeded in creating a unique ultrafine polycaprolactone electrospun material and in applying it in a model of complicated anastomotic healing on the pig colon. The planar PCL layer was fabricated via needleless electrospinning technique, a method suitable for eventual large-scale production. The material is easy to use without any need for further fixation. The presence of the material did not cause any adverse effects in vivo. The PCL layer showed good cytocompatibility and biocompatibility and was well tolerated during the whole animal study. The material is also not pro-adhesive and did not cause anastomotic strictures or other complications. The anastomotic specimens showed significantly higher levels of collagen after the 3 weeks of observation, which is an indirect sign of higher mechanical strength. Impact on the risk of AL was not observed directly as no AL appeared in either group. We intend to develop new versions of the material with active agents and study them further in adjusted experimental settings to obtain more distinguishable results before moving to clinical studies on colorectal surgical patients.

## Data Availability

All data included in the article or supplementary materials.

## Supplementary Materials

The following are available online at https://www.mdpi.com/2227-9059/9/2/102/s1, Figure S1: Nanospider scheme, Figure S2: Perianastomotic adhesions amount scoring system, Table S1: Leukocytosis development, Document S1: power analysis.

## Appendix A

**Table A1:** Process parameters of the needless electrospinning via Nanospider^TM^.

Distance between the electrodes [mm]	175
Voltage Electrode 1 [kV]	−10
Voltage Electrode 2 [kV]	40
Rewinding speed [mm/min]	60
Cartridge movement speed [mm/s]	450–500
Temperature [°C]	22
Relative humidity [%]	50

## Appendix B

**Table A2:** Intestinal Wall Integrity Score.

Layer	Points	Finding
Mucosa	1/4	Completely re-epithelized
	0/4	Incompletely re-epithelized
Submucosa	1/4	Completely healed
	0/4	Purulent infiltration, necrosis
Muscularis *	3/12	No distance (≤0.09 mm)
	2/12	Distance 0.1 to 1.99 mm
	1/12	Distance 2 to 3.99 mm
	0/12	Distance over 4 mm
Serosa	3/12	No purulent infiltration and necrosis
	2/12	Purulent infiltration and/or necrosis from the muscular layer to area of nanomaterial **
	1/12	Purulent infiltration and/or necrosis from the area of nanomaterial to the peritoneum ***
	0/12	Purulent infiltration and/or necrosis passes to the peritoneum

* Distance between the two anastomosed muscle layers. ** 2/12 points for purulent infiltration and/or necrosis from muscular layer to half thickness of the serosa in the Control group. *** 1/12 points for purulent infiltration and/or necrosis from half thickness of the serosa to the peritoneum in the Control group.

## Appendix C

**Table A3:** Weight profile of experimental animals.

Animal	POD 0 Weight (kg)	POD 3 Weight (kg)	POD 7 Weight (kg)	POD 14 Weight (kg)	POD 21 Weight (kg)
Control group					
cg 01	34	31.6	31.4	33.8	33
cg 02	31	30.1	29.4	30.7	30.7
cg 03	37	33.8	33.7	34.5	34.5
cg 04	45.5	41.3	41.1	42	42.1
cg 05	48.4	43.6	42.8	44	43.8
cg 06	30.6	30	30.7	29.5	32.5
cg 07	30	29	29.8	33.6	32.1
cg 08	27.8	25.9	27.2	30.5	30.5
Experimental group					
eg 01	28.7	28.3	29.2	29.4	29.4
eg 02	29.9	29.3	28.2	29	30.3
eg 03	35.8	35.2	35.2	39	37.9
eg 04	37.3	36.8	36	41	41
eg 05	41.7	41.5	41.3	43.4	45.2
eg 06	42.9	42.2	42.2	43.8	45.1
eg 07	27.9	27.4	26.3	29.4	30
eg 08	27.2	26.8	25.8	29.1	29
