# Supplementary material for: Reinforcement of Colonic Anastomosis with Improved Ultrafine Nanofibrous Patch: Experiment on Pig
Source: Biomedicines. 2021 Jan 21;9(2):102. doi: 10.3390/biomedicines9020102 (PMC7909771; doi:10.3390/biomedicines9020102)
Supplement: Supplementary file 1 [file biomedicines-09-00102-s001.zip › Figure S2.docx]

**Standard operating procedure**

**Title:** Perianastomotic adhesions amount scoring system

**Created by:** Rosendorf J., Biomedical Center, Faculty of Medicine in Pilsen, Charles University, Czech Republic; verified by Liska V. (Head of the laboratory)

**Date of creation:** 10/2017, translated to English 10/2019

**Purpose:** Created for the project ‘Experimental fortification of intestinal anastomoses with nanofibrous materials in a large animal model’. Project code: MSMT-26570/2017-2. The aim of this protocol is standardization of sample collection and documentation. The experimental animals are subjected to laparotomy in full anesthesia prior to this procedure.

**Steps:**

1. Take a picture of each anastomosis in situ as well as of the whole abdominal cavity to be archived.
2. Release each anastomosis from peritoneal adhesions if present; leave as much adhesion tissue as possible on the intestine while doing so.
3. Evaluate the quality of adhesions while performing the step number 2 according to Zühlke’s grading system for peritoneal adhesions:
   - Grade 0: no adhesions
   - Grade 1: filmy adhesions easy to separate by blunt dissection
   - Grade 2: blunt dissection is possible for the most of adhesions but some sharp dissection is needed, beginning vascularization
   - Grade 3: adhesiolysis is possible by sharp dissection, clear vascularization and bleeding from adhesions during sharp dissection
   - Grade 4: adhesiolysis is possible by sharp dissection but is very challenging. Organs are strongly attached and damage is hardly preventable.
4. Resect each anastomosis together with 2 cm of oral and aboral intestine.
5. Cut the collected sample of intestine immediately after collection longitudinally along the mesentery. Treat the specimens carefully.
6. Pin the sample to a cork underlay. Mucosa faces the cork. Oral part faces a marked side of the cork underlay.
7. Cut a V-shaped mark into the side of the cork underlay where the oral part of the specimen is.
8. Assign a random alphanumeric code to the anastomosis sample.
9. Take a picture of the anastomosis on the cork underlay with both animal code/number of anastomosis (1/2/3 starting orally) and the new alphanumeric code to be archived.
10. Measure the specimen and divide its circumference into four parts along the suture line. Assign to each quadrant (starting from left to right to respect the polarity of the intestine) a number of points according to the extent of adhesions (Fig. 1):
    - Zero points for no adhesions in the segment
    - One point for a segment being partially covered by adhesions
    - Two points for a fully adhered segment
11. Put the samples of the intestine into 300 ml storing containers filled with10 % buffered formalin. Mark the containers with the newly created alphanumeric code (for blinding during the histologic evaluation).


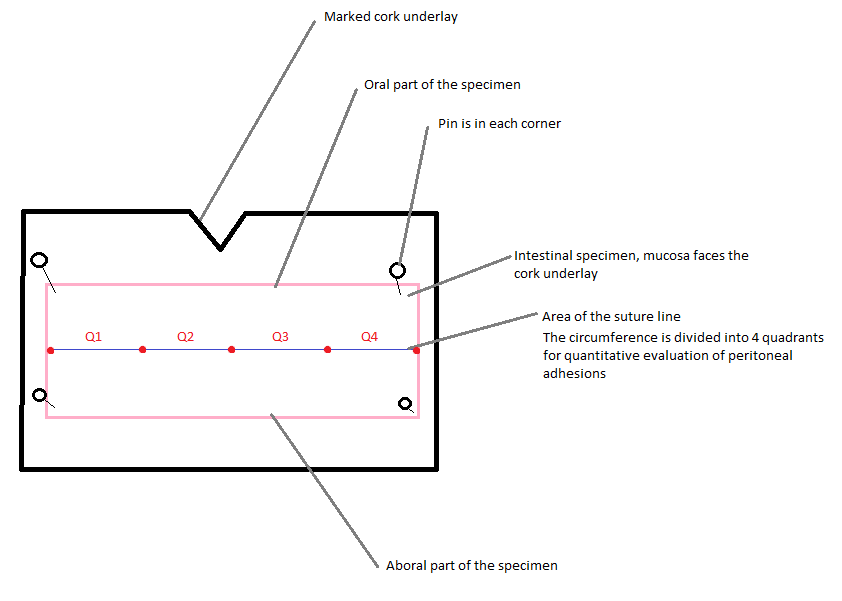


*Fig. 1: Scheme of the specimen of the intestine pinned to a cork underlay, divided into four quadrants (Q1-Q4)*
