# Supplementary material for: Reinforcement of Colonic Anastomosis with Improved Ultrafine Nanofibrous Patch: Experiment on Pig
Source: Biomedicines. 2021 Jan 21;9(2):102. doi: 10.3390/biomedicines9020102 (PMC7909771; doi:10.3390/biomedicines9020102)
Supplement: Supplementary file 1 [file biomedicines-09-00102-s001.zip › Figure S1.docx]

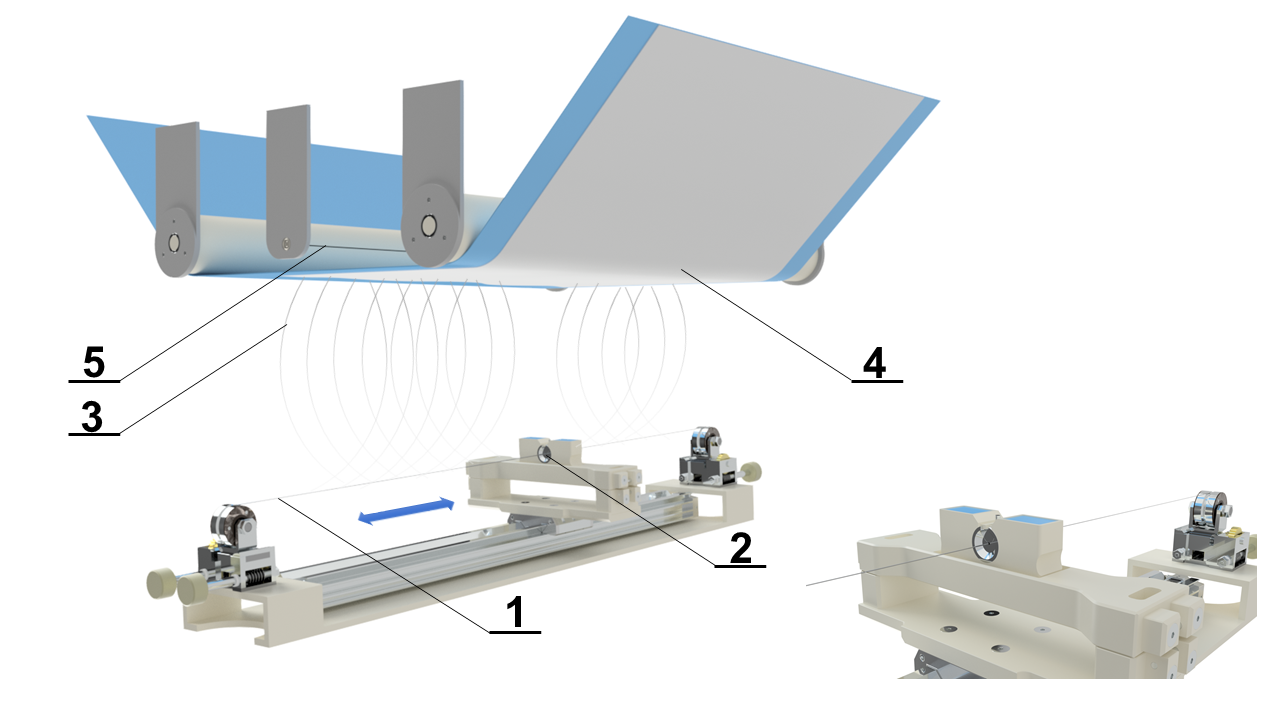
 *Figure S1*: Nanospider^TM^ needleless electrospinning device: 1 – steel wire serves as the positive electrode, 2 – steel orifice with a reservoir for polymeric solution, 3 – nanofiber formation, 4 – created nanofibers are collected on the spunbond substrate, 5 – negative electrode.
