# Supplementary material for: Reinforcement of Colonic Anastomosis with Improved Ultrafine Nanofibrous Patch: Experiment on Pig
Source: Biomedicines. 2021 Jan 21;9(2):102. doi: 10.3390/biomedicines9020102 (PMC7909771; doi:10.3390/biomedicines9020102)
Supplement: Supplementary file 1 [file biomedicines-09-00102-s001.zip › power analysis.docx]

# Sample size estimation

1. We decided to construct one intestinal anastomosis with artificial defect per animal.
2. We assumed the same sample size for both experimental and control group.
3. We considered the Intestinal Wall Integrity Score (IWIS) as the most valuable result of the study. We anticipated a very low IWIS of 30% in the Control group with an uncovered anastomotic defect and a substantially higher IWIS of 60% in the Experimental group as an effect of our nanomaterial patch. Standard deviation of 20 percentage points was assumed in both groups.
4. After performing power analysis for two-tailed two-sample t-test, sample sizes of 7 (1 anastomosis x 7 animals, test power 73.13%) and 8 (1 anastomosis x 8 animals, test power 79.65%) were considered. Finally, the power close to 80% provided by the 8-animal group size was preferred.
